# Orientation Matters: Polarization Dependent IR Spectroscopy of Collagen from Intact Tendon Down to the Single Fibril Level

**DOI:** 10.3390/molecules25184295

**Published:** 2020-09-19

**Authors:** Gorkem Bakir, Benoit E. Girouard, Richard Wiens, Stefan Mastel, Eoghan Dillon, Mustafa Kansiz, Kathleen M. Gough

**Affiliations:** 1 Department of Chemistry, University of Manitoba, Winnipeg, MB R3T 2N2, Canada; bakirg@myumanitoba.ca (G.B.); girouar3@myumanitoba.ca (B.E.G.); umwiensr@gmail.com (R.W.); 2 neaspec GmbH, Eglfinger Weg 2, 85540 Munich-Haar, Germany; Stefan.Mastel@neaspec.com; 3 Photothermal Spectroscopy Corp., 325 Chapala St, Santa Barbara, CA 93101, USA; eoghan@photothermal.com (E.D.); mkansiz@photothermal.com (M.K.)

**Keywords:** collagen type I, tendon, fibrils, far-field infrared spectroscopy, optical photothermal spectroscopy (O-PTIR), scattering-type scanning near-field optical microscopy (s-SNOM), nano-FTIR spectroscopy, polarization imaging and spectroscopy, advances in IR imaging

## Abstract

Infrared (IR) spectroscopy has been used for decades to study collagen in mammalian tissues. While many changes in the spectral profiles appear under polarized IR light, the absorption bands are naturally broad because of tissue heterogeneity. A better understanding of the spectra of ordered collagen will aid in the evaluation of disorder in damaged collagen and in scar tissue. To that end, collagen spectra have been acquired with polarized far-field (FF) Fourier Transform Infrared (FTIR) imaging with a Focal Plane Array detector, with the relatively new method of FF optical photothermal IR (O-PTIR), and with nano-FTIR spectroscopy based on scattering-type scanning near-field optical microscopy (s-SNOM). The FF methods were applied to sections of intact tendon with fibers aligned parallel and perpendicular to the polarized light. The O-PTIR and nano-FTIR methods were applied to individual fibrils of 100–500 nm diameter, yielding the first confirmatory and complementary results on a biopolymer. We observed that the Amide I and II bands from the fibrils were narrower than those from the intact tendon, and that both relative intensities and band shapes were altered. These spectra represent reliable profiles for normal collagen type I fibrils of this dimension, under polarized IR light, and can serve as a benchmark for the study of collagenous tissues.

## 1. Introduction

The infrared (IR) spectrum of type I collagen has been studied for decades [1,2,3,4,5,6,7]. The importance of collagen in the health and maintenance of the human body is extensive; the hierarchical structure and radial symmetry of collagen render it a suitable target for study with polarized IR light, for example: type I collagen [1,2,6,7,8] and type II collagen [9]. The dichroic behavior of ordered collagen fibrils was first demonstrated with IR spectro-microscopy nearly 70 years ago [1]. We have studied collagen in scar tissue [10,11] and in mechanically damaged tendon [7], where we exploited IR polarization imaging to probe the degree of disorder in the damaged tissue.

A limitation to the IR spectroscopic analyses of collagen has been the lack of definition of the spectral profile that constitutes a molecular level of order in the normal control tendon and fibrils. Typically, the IR polarization contrast is defined chiefly by changes in the relative intensity of the Amide I (primarily carbonyl stretch) and Amide II (primarily C-N backbone stretch with some C-N-H angle bend) vibrational bands. In fact, numerous bands in the complicated spectrum of highly ordered collagen type I respond differently under polarized infrared light [1,2,7,9,12]. Our previous work was primarily based on far-field Fourier transform IR (FF-FTIR) imaging with focal plane array (FPA) detection, which is subject to the usual Rayleigh criterion resolution limits for λ = ~7 μm wavelength, corresponding to the main protein IR absorbance bands, Amide I and II [7]. We then began using nanoscale FTIR (nano-FTIR) spectroscopy employing the scattering-type scanning near-field optical microscopy (s-SNOM) method [13,14,15,16,17], wherein the diffraction limit is circumvented and IR spectra may be obtained with a spatial resolution of ~20 nm, far below the wavelength of the IR radiation (typically 5–10 μm). Another complementary method is optical photothermal IR (O-PTIR) imaging and spectroscopy [18,19,20], a relatively new far-field technique that opens a new window onto the study of collagen, both as intact tissue and as fibrils, with IR wavelength-independent resolution of better than 500 nm.

In this paper, we seek a better understanding of the absolute variation in spectra of ordered collagen, as this can improve the evaluation of disorder in damaged collagen in the future. To this end, we employed multiple techniques that provide a variety of polarized IR sample illumination and spatial resolutions to investigate the dependence of collagen IR spectra on these parameters. Polarized IR spectra and images of intact, mechanically aligned tendon were obtained with FF-FTIR equipped with focal plane array (FPA) detection and with O-PTIR. To polarize the IR radiation in the FF-FTIR measurements, we inserted a polarizer in the path of the IR beam, after passage through the sample; the lasers employed in O-PTIR are inherently linearly polarized. It was also possible to obtain spectra of control, unstretched fibrils with nano-FTIR spectroscopy, and of similar fibrils with a quantum cascade laser (QCL) oriented with (parallel) and across (perpendicular) the fibril direction by O-PTIR. The nano-FTIR spectra are necessarily polarized with respect to the probe tip axis, and thus yield spectra perpendicular to the fibril direction only. The results from the intact tendon (FF-FTIR and O-PTIR) are contrasted with those from sub-micron fibrils (O-PTIR and nano-FTIR), in an effort to obtain a clearer understanding of the variations observed and their relation to internal structural order.

## 2. Results

Infrared spectra have been acquired from intact bovine positional tendons and from fibrils extracted from these tendons. The intent is to compare the spectral profiles that appear under conditions of polarized IR illumination using diverse instrumental approaches. The sample preparation for the intact tendon and fibrils should leave the tendon free of possible contamination, whether phosphate buffer solution (PBS) or optimal cutting temperature (OCT) cryoprotectant (see Methods). In one case only (O-PTIR spectra of fibrils), some OCT was observed; this was easily removed by spectral subtraction.

### 2.1. Far-Field IR Spectroscopy of Intact Tendon with Polarized Light

#### 2.1.1. IR Polarization Contrast FTIR Microscopy with Focal Plane Array Detection

**Figure 1:**
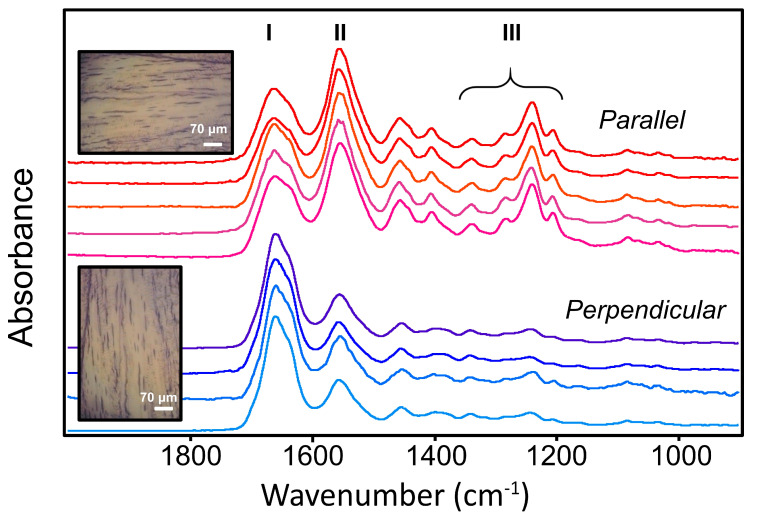
Far-field Fourier transform IR (FF-FTIR) spectra of intact tendon on BaF_2_ from mosaic images obtained with focal plane array (FPA) detection, labelled to show Amide I, II and III regions. Each spectrum is derived from a different mosaic and is an average of about 20 spectra selected from the most oriented region in the field of view. Visible images show typical appearance of straightened tendon, imaged with same polarizer setting (90°) aligned with or rotated to be perpendicular to the polarizer. Scale bar = 70 μm.

In the course of our study on mechanically damaged tendons [7], we acquired a multitude of polarization contrast images of tendon sections. Here, we have drawn on our library of data to present spectra representative of collagen that is intact, and undamaged (Figure 1). In each case, an FTIR FPA image, comprising several thousands of spectra, was examined, and some 20 spectra extracted that met the criteria of similar high-quality signal-to-noise ratio (SNR), level baseline and absence of scattering artifacts. In addition, the spectra were selected only from regions of the image where the polarization contrast and visible record showed that the normally crimped tendon had been drawn straight, without rupture or introduction of kinks [7].

The spectra exhibit the expected profiles of type I collagen, with a slightly asymmetric Amide I, and profound differences in the relative intensities of most bands under IR light polarized parallel (Figure 1, upper set) or perpendicular (Figure 1, lower set) to the orientation of the fibers in the tendon. Samples were imaged either with a single orientation under two different polarizer settings (0° and 90°) relative to the fiber orientation, or with polarizer set at 90° and sample rotated by 90°, to test for any instrumental bias, as shown in the white light images. Results were consistent regardless of procedure.

We observe a slight narrowing of the full width at half maximum of the Amide I in the perpendicular orientation, 60 (SD = 2) cm^−1^, compared to parallel, 74 (SD = 3) cm^−1^, which is in part due to the contribution of the much stronger Amide II band in the parallel orientation. Rather than attempt to quantify these variations at this stage, we next compare the results with those from other methodologies that yield data from smaller voxels.

#### 2.1.2. IR Polarization Contrast Spectroscopy with Optical-Photothermal IR (O-PTIR) Detection

**Figure 2:**
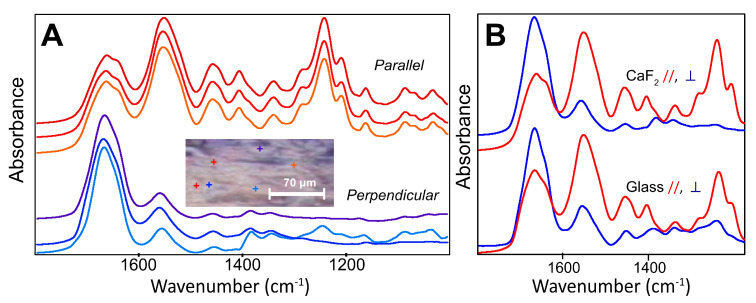
Optical photothermal IR (O-PTIR) spectra from intact tendon, from ~500 nm measurement spots. (**A**) Individual spectra obtained from the two orientations of a section mounted on a CaF2 window, relative to the linearly polarized quantum cascade laser (QCL). Inserted visual image shows the 6 locations, all of which lie within the region imaged with FTIR FPA; scale bar = 70 μm. Colored + correspond to spectral colors. (**B**) Comparison of spectra obtained from CaF2 (top) and glass (bottom) substrates in parallel and perpendicular orientations to linearly polarized QCL.

Vibrational spectra were collected with the mIRage O-PTIR microscope (Photothermal Spectroscopy Corp.) from single points (~500 nm) in a region of oriented tissue in one of the sections mounted on a CaF_2_ window and shown in Figure 1. This O-PTIR operates on the principle of photothermal detection [18,19,20], in which an IR quantum cascade laser (QCL) excites the sample’s molecular vibrations in the spectral range of 1800–900 cm^−1^; the resultant photothermal effect is detected via a short wavelength probe laser. As the IR lasers are highly linearly polarized, the spectra are expected to be comparable to a great extent with those obtained from the FTIR microscope with IR polarizer. In order to obtain parallel and perpendicular spectra, the samples were oriented to be aligned with, or rotated to be perpendicular to, the laser polarization. The results for the tendon on CaF_2_ and on glass are shown in Figure 2.

The spectra in Figure 2A,B show that the high contrast achieved with the inherent laser polarization yields spectra that are very similar to those obtained from the first experiments with FTIR FPA and polarizer. Similar results were obtained for the serial section mounted on a glass microscope slide, and spectra were obtained with equally good SNR quality (Figure 2B).

### 2.2. Far-Field IR Spectra of Collagen Fibrils with Polarized Light

It was not possible to obtain a spectrum from a single fibril with the FF-FTIR FPA, despite a very high accumulation of scans (2048 for background and 512 for sample) with the high magnitude optics engaged, even without the polarizer in place. Inclusion of the polarizer dropped the SNR for the FF-FTIR FPA by more than one half. This result is not surprising, given that the fibril dimension is an order of magnitude below the diffraction limit of FF-FTIR and there is very little material. Thus, this approach could not reasonably be used on single fibrils. Spectra of individual fibrils, extracted from control tendon in the same manner as described in our previous work [7], were successfully obtained by O-PTIR and by nano-FTIR spectroscopy instruments

#### 2.2.1. IR Polarization Contrast Spectroscopy of Isolated Fibrils with O-PTIR

**Figure 3:**
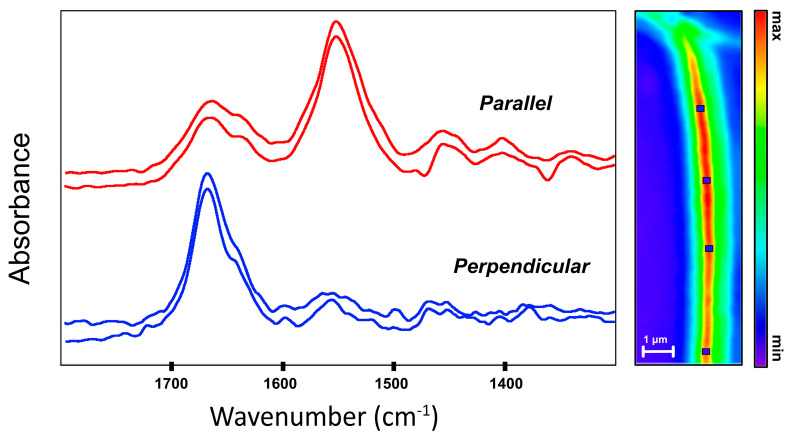
Spectra obtained with O-PTIR from control tendon fibrils on CaF_2_ window. Top spectra (red) obtained with fibril parallel to laser polarization; bottom spectra, sample rotated by 90°. Single frequency image at right recorded at 1655 cm^−1^ in perpendicular orientation. Squares denote locations at which some of the spectra were acquired. Scale bar = 1 μm.

O-PTIR spectra were obtained from numerous points along a control fibril prepared from a tendon segment that was not subjected to mechanical damage, (Figure 3). Once identified, the same fibril was examined with the orientation horizontal (parallel) and vertical (perpendicular) to the IR laser polarization, by rotating the sample on the stage, as was done for the intact tendon. Traces of OCT medium were found in all spectra. The fibrils had been extracted from a piece of tendon that had originally been encased in OCT medium and, evidently, removal of this medium by several sequential washings in ultrapure water was insufficient to eliminate it. A single FF-FTIR FPA image of the droplet’s center was taken later (data not shown), and confirmed the contaminant identity as OCT. The spectra in Figure 3 were taken furthest from the concentrated OCT region and were corrected by subtraction of the OCT spectrum. Signature bands of collagen from 1400 to 900 cm^−1^ were evident but obscured by uneven OCT contamination. Fortunately, OCT does not absorb across the Amide I and II region, allowing a clean window onto those modes. The O-PTIR SNR was estimated to be 40 for parallel and 70 for perpendicular orientations based on the signal maximum for the strongest band and the peak to peak noise between 1795 to 1750 cm^−1^ in each case.

The Amide I and II profiles are much as expected: in the perpendicular orientation the Amide I dominates, while the Amide II is strongest in the parallel orientation, as seen in intact tendon. Both bands were narrower than those recorded from intact tendon; this observation is discussed below.

The fibril was imaged with O-PTIR at several single frequencies, to get a confirmatory estimate of the apparent physical width. The intense red-yellow band from the single frequency image recorded at 1655 cm^−1^ in perpendicular orientation (Figure 3, right side) shows this fibril to be no more than 500 nm across, based on the line profile of intensity across the fibril (data not shown). This dimension qualifies the target as a true fibril and is comparable to the 300 nm fibril examined for the first IR s-SNOM experiments [7].

#### 2.2.2. IR Polarization Contrast Spectroscopy with Nano-FTIR Spectroscopy Detection

**Figure 4:**
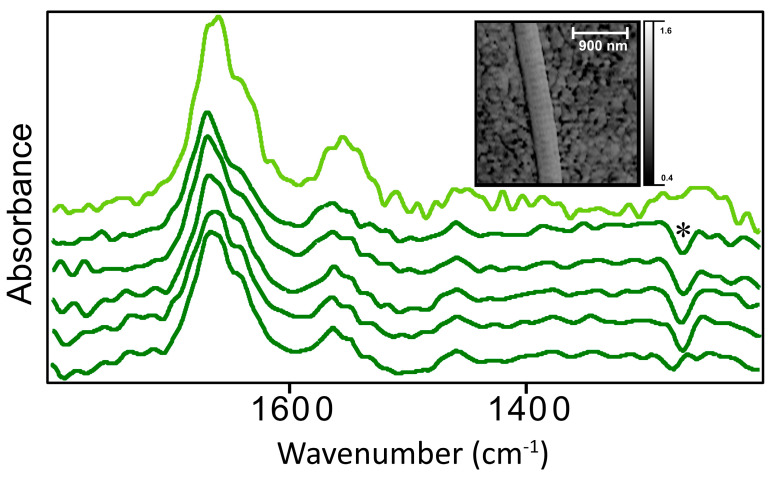
Nano-FTIR spectra of control tendon fibrils. Top light green spectrum is the average of 19 spectra acquired synchrotron light at the Advanced Light Source. Bottom five spectra (dark green) were acquired with a neaSNOM microscope (neaspec GmbH, Munich-Haar, Germany). Asterisk (*) at 1265 cm^−1^ shows negative peak due to polydimethylsiloxane (PDMS) contamination of the AFM tip. Inserted AFM image shows a typical control fibril, lying on sputtered Au surface, with D-banding; scale bar = 900 nm.

As part of an on-going study, we have been collecting nano-FTIR spectra on control and mechanically damaged collagen fibrils at the Advanced Light Source, LBNL, Berkeley CA, USA. One of the motivations for the present analysis of control fibrils is to gain a clearer understanding of the differences between control and damaged fibrils. Earlier studies showed that the nano-FTIR method favors absorption bands that are aligned with the Atomic Force Microscopy (AFM) tip [7]. This effect, which was expected [15], arises from the tip-sample polarization of the electric field; however, prevents acquisition of spectra parallel to a fibril lying on the AuSi substrate. We plan to exploit this feature to identify molecular scale changes in damaged fibrils. Spectra obtained with nano-FTIR spectroscopy, from control fibrils that were not subjected to mechanical damage, are shown in Figure 4.

The AFM image in Figure 4 illustrates the normal D-banding in an isolated control fibril, one of the series that were examined. Five spectra were obtained with laser illumination (neaspec GmbH, Munich-Haar, Germany) at points on a fibril previously examined at BL 2.4 Advanced Light Source (ALS) with the neaSNOM microscope and synchrotron illumination. The SNR for spectra obtained at ALS is always weaker than that obtained with nano-FTIR lasers. A total of 19 single point spectra taken from control fibrils at ALS were summed to give the ALS spectrum shown here. The negative polydimethylsiloxane (PDMS) band at 1265 cm^−1^ is noted in some of the spectra in Figure 4 (see Methods). Its presence interferes with the detection of the Amide III series of bands (1337, 1284, 1240 and 1204 cm^−1^) but these would not be prominent in the perpendicular orientation.

Significantly, nano-FTIR spectroscopy allows us to record very good spectra on a single fibril; the signal quality is slightly lower due to the small probing volume. For nano-FTIR spectroscopy, and IR s-SNOM in general, the volume is estimated as (~20 nm)^3^, compared to other techniques, (500 nm)^3^ for FF O-PTIR on fibrils, (500 nm)^2^ × (2 to 3 μm) for FF O-PTIR on intact tissue, and ~3 μm × 3 μm × 5 μm or ~45 μm^3^ for conventional FF-FTIR FPA spectroscopy, adjusted for the IR wavelengths of the Amide I and II bands and tissue depth ~5 μm.

### 2.3. Comparison of Spectra from All Methods

**Figure 5:**
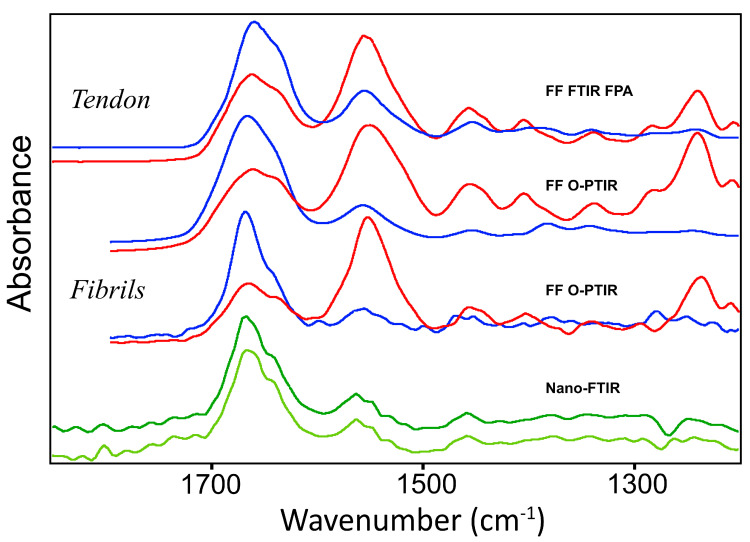
Averaged spectra from each experimental procedure, offset for clarity. As in other figures, all spectra with polarization parallel to fiber or fibril direction are shown in red; perpendicular are blue. Intact tendon spectra from FF-FTIR FPA and O-PTIR are sums of spectra appearing in Figure 1, Figure 2 and Figure 3. The nano-FTIR spectra at the bottom represent sums of three separate experiments, two with lasers (dark green) and the sum of 19 spectra from ALS, bottom-most spectrum (light green).

Figure 5 summarizes the data above. All spectra were collected as a single average for each orientation and instrument.

## 3. Discussion

Collagen molecules in a positional tendon are aligned in an inherently organized, hierarchical manner, thus tendon is eminently suitable for analysis with polarized IR light [1]. While polarization studies with other methods are common, few have used polarized IR spectroscopy [1,2,7,9,12]. IR wavelength-independent spatial resolution FF O-PTIR and nano-FTIR technologies are rapidly advancing our ability to collect IR spectra at spatial resolutions below the IR diffraction limit. Our motivation in this work has been to achieve a deeper understanding of what the different experimental approaches can reveal for type I collagen, in order that we can better evaluate mechanically damaged tissue and ordered/disordered scar tissue, where the organization is significantly altered.

Early IR polarization studies of collagen quickly addressed the complexity of dichroism in collagen [2,3], with recognition that the direction of the change of the molecular dipole moment in the Amide modes would not be precisely parallel to the orientation of the bonds. Some band assignments have been achieved through analysis of synthetic biopolymer mimics [21] and temperature-dependent studies [4]. The Amide I mode is generally referred to as a C=O stretch, but vibrational energy and alignment of the dipole moment change depending on the amide orientation. The Amide II is mostly due to C-N stretch with some C-N-H angle bend, but the fundamental mode could well be nearly perpendicular to the direction of the N-H bond. The presence of other amino acid residues, and unknown quantities of water of hydration, lead to further unresolvable spectral contributions even in pure collagen.

The hierarchical organizational structures in tendons are loosely classified by their diameter as fiber (2000–500 nm), fibril (500–100 nm) and sub-fibril (<100 nm) diameter, while the diameter of an individual collagen triple helical molecule is about 1.5 nm. Given the mode of self-assembly, it is not realistic to define these more precisely [22,23]. As we showed previously [7], actual rupture results in a mixture of orientations on a micron spatial scale, producing spectra that are intermediate between the primarily parallel and perpendicular extremes. Some variation in orientation must exist simply because of tissue depth, as those tendons were cryosectioned at 5 and 8 μm thickness. Intact tendons contain additional non-collagenous components that help to maintain the gliding functions, as well as cells, such as fibroblasts and synovial cells [22]. The absence of a lipid carbonyl band at 1735–1740 cm^−1^ and of the symmetric CH_2_ stretch band (data not shown) shows that we are not detecting any significant amounts of cellular material. These bands were detected in some spectra of intact tendon, and for this reason were not selected for the data presented here.

The spectral information shown in Figure 1 has spatial resolution that is IR wavelength dependent. The volume probed with the FF-FTIR FPA microscope and high magnification option is at best ~1.1 μm × 1.1 μm × 5 μm [24] when applied to bands at short wavelengths, such as 3 μm for NH and CH stretch modes. For the Amide bands considered here, the Rayleigh criterion spatial resolution is ~4 μm, with 1.1 μm pixel size oversampling [24,25,26], and the probed volumes are estimated to be on the order of 3 μm × 3 μm × 5 μm. In comparison with the fibril spectra below, the Amide bands are relatively broad, as can be expected for the heterogeneity present, even though the tendons had been stretched sufficiently to eliminate normal crimping. The spectra show the expected profiles under parallel and perpendicular polarization, wherein the distinct polarization contrast permits assessment of relative tissue organization at the micron length scale.

The application of O-PTIR for analysis of oriented materials was recently demonstrated for a cross-section of a commercial plastic bottle [19]. The plastic wall was 350 μm thick, thus it was possible to obtain polarized spectra with an estimated pixel resolution of 500 nm across the wall thickness. Linear dichroism was observed in the spectra for polyethylene terephthalate PET backbone methylene wagging modes, in contrast to C=O stretch modes, which were anticipated to be mainly perpendicular to the chain direction. Here, we present the first example of O-PTIR polarized spectroscopy of a biopolymer, collagen.

From the FF O-PTIR spectra of intact tendon (Figure 2A), we can conclude that the polarization of the lasers in the mIRage microscope give results that are essentially equivalent to those obtained with a thermal source FF-FTIR FPA and IR polarizer. The SNR is excellent, particularly on noting that, for single points at least, the data collection time was 30 s under our operating conditions. The band profiles reveal further differences in the Amide bands. The low energy shoulder in the Amide I under parallel polarized light is even more clearly defined with O-PTIR. It is still apparent, though greatly diminished, in the perpendicular orientation. The depth penetration of the O-PTIR spectra is not precisely definable, though presumably a few microns, and possibly the entire depth of these dried sections as it depends on the material probed; however; the spot size is ~500 nm and the spectrum must be more localized than that of FF-FTIR FPA.

Interestingly, relative intensities of the Amide I and II bands in O-PTIR differed slightly more than was observed with the FPA, as did the contrasting profiles within the Amide I. This is possibly due to a higher IR polarization contrast with the QCL, though this seems unlikely, as the IR polarizer is very efficient. Instead, the higher spatial resolution afforded by O-PTIR may probe a more purely oriented local section of the tendon fibrils. The FF-FTIR gives an average of the chemistry from within a few to several micron diameter voxels, while with better spatial resolution, the submicron heterogeneity may be clearer.

The O-PTIR signal intensity was not dependent on the substrate, as numerically comparable results were obtained from the section on glass compared to that on CaF_2_, under the same measurement conditions and sample orientation (Figure 2B). Point to point variation was no greater than that typically observed under FF-FTIR and is ascribed to slight variations in tissue thickness or condition. The ability to measure samples on standard glass slides, without the loss of signal below 1600 cm^−1^, as occurs with transmission FTIR on salt windows, is another key advantage of using O-PTIR. The sample may be stained after spectra have been recorded; the glass substrate is inexpensive and is a normal part of histology workflow.

The profiles for the Amide bands of fibrils obtained with O-PTIR (Figure 3) are similar to those of intact tendon obtained with O-PTIR (Figure 2) but with cleaner and more pronounced differences. The spectra parallel to the fibril length exhibit a strong Amide I shoulder at lower energy that is nearly absent in the perpendicular spectrum. The increased intensity of the main Amide I absorbance, centered around 1655–1660 cm^−1^ could be masking the shoulder but relative intensities of individual bands under different polarizations cannot be ascertained.

Owing to the radial symmetry of the collagen fibril, and the high polarization in the z-direction (parallel to AFM tip), the nano-FTIR spectra of collagen fibrils are expected to be equivalent to spectra acquired perpendicular to the fibril direction, in the sample x-y plane. Spectra in Figure 4 are very similar to those observed in our previous work [7], whether acquired with synchrotron IR light, or the nano-FTIR laser system.

Direct comparison of the averaged spectra from intact tendon and from fibrils, from all experimental techniques (Figure 5), shows clearly that the Amide bands from intact tendon spectra are considerably broader than those obtained from fibrils, regardless of whether intact tissue spectra were obtained by FF-FTIR FPA or FF O-PTIR. The relative intensity of Amide II to Amide I band drops from about 1.3 (parallel) to 0.3 (perpendicular).

Owing to the sample dimension and sub-fibril heterogeneity [22], it is not possible to rationally resolve the multiple contributions within either band. It is important to note that, historically, procedures such as curve-fitting, second derivatization (2ndD), and Fourier self-deconvolution (FSD), have sometimes been applied to spectra in an effort to resolve overlapping bands [4]. We applied FSD and 2ndD to the spectra of intact tissue obtained with FF-FTIR FPA and FF O-PTIR. FSD parameters are typically left to the operator choice; we used a bandwidth of 16 cm^−1^ and enhancement factor of 2. The pseudo-deconvolved spectra bore little resemblance to the fibril spectra acquired with sub-micron techniques: relative intensities did not match, multiplets appeared at wavelengths where no bands appeared in the fibril spectra (data not shown). It might be argued that these bands could reflect multiple oblique fibril orientations within the tissue thickness, but these are not unique solutions and cannot provide the information that we sought. There can be no substitute for data acquired at genuinely wavelength-independent submicron spatial resolution.

Comparisons among all spectra show that Amide bands taken with IR light parallel or perpendicular to the control fibril direction are much narrower than those from intact tendon. This indicates that the fibril target is more uniform, whether assessed at ~500 nm (O-PTIR) or 200 nm (nano-FTIR spectroscopy). These dimensions represent the typical range of the “fibril” width; thus, it is very satisfying to see the similarity.

The Amide I band is sharply narrowed in the perpendicular polarized spectra from either method. The principal contribution is from the higher energy band centered at ~1660 cm^−1^. The ratio of the Amide II to Amide I integrated band areas is still about 0.3; however, this now reflects the dominant higher energy component. The lower energy shoulder at 1635 cm^−1^ is generally thought to arise from the glycine C=O, which points outward from the triple helix, and is mostly available for H-bonding with water [3,4]. Such H-bonding would weaken the C=O bond and lower the vibrational energy. If so, then the spectra can be interpreted to mean that this carbonyl is generally aligned more with the fibril direction. Only the O-PTIR technique allows for spectra with polarization parallel to the direction of an individual fibril. The intensity of the 1660 cm^−1^ band drops significantly, while that of the lower energy band at 1635 cm^−1^ remains distinct. This observation is in keeping with the perpendicular spectra, in that a band more aligned with the fibril direction (1635 cm^−1^) should remain strong when the polarization is rotated to the parallel. The triple helix axis is tilted by up to 15 degrees with respect to the microfibril axis (bundle of five collagen triple helices where the molecules are axially quarter-staggered), based on X-ray scattering data on tendons [22]. Given the dimensions of the fibrils, we must assume this amount of variation is present. The absence of a lipid carbonyl band at the 1735–1740 cm^−1^ region confirms that there is little, if any, cellular debris remaining, and that these are spectra of clean, normal D-banded, undamaged collagen fibrils. The O-PTIR and nano-FTIR spectra of fibrils give confirmatory results for spectra with polarization perpendicular to the fibril axis, while the O-PTIR also enables the complementary result for polarization parallel to the fibril axis. We conclude that these spectra represent reliable profiles for control collagen type I fibrils of this dimension, under polarized IR light, and will serve as benchmark references for future studies.

## 4. Materials and Methods

Tendon samples came from a series described previously [7]; preparation is summarized briefly here. Tendons were dissected from the tails of young adult steers killed for food, and immediately stored at 4 °C in phosphate buffered saline (PBS) containing 1% antibiotic/antimycotic solution (product A5955, Sigma-Aldrich, Oakville, ON, Canada). Prior to the mechanical damage procedure, a 15-mm-long sample was removed from each tendon to serve as an unloaded control. Using a servo-hydraulic material testing system, the remainder of each tendon was subjected to 5 cycles of sub-rupture overload at a strain rate of 1%/s, as described previously [7,27]. Control and stretched tendon segments, approximately 10 mm long by 4 mm diameter, were stored at room temperature for 24 h in PBS containing 1% antibiotic/antimycotic solution and 1% protease inhibitor (product S8820, Sigma-Aldrich, Oakville, ON, Canada); shipped overnight to the University of Manitoba, where they were immediately removed from the PBS medium, covered with OCT (Sakura Finetek Inc., Torrance, CA, USA), frozen in isopentane-cooled in liquid N_2_, and stored at −80 °C. Cryosections were cut to 5 or 8 μm thickness at −21 °C, and mounted on CaF_2_ or BaF_2_ salt windows, as well as on glass microscope slides (for O-PTIR).

For fibril analyses, a thawed control tendon segment was washed as follows: the thawed segment was rinsed in ultra-pure water to remove the majority of the OCT; it was then transferred to a 1.5 mL Eppendorf with ultra-pure water, soaked for 20 min, rinsed again and transferred to a fresh Eppendorf.; this was repeated 6 times. The rinsed segment was dissected further using tweezers and a fine-tipped glass rod in 1 mL of ultra-pure water until the mixture appeared cloudy, releasing many suitable fibrils [28]. Droplets of the suspensions were deposited on CaF_2_ salt windows or on 1 cm^2^ gold-coated silicon wafers for O-PTIR and nano-FTIR analysis, respectively.

For FF-FTIR, tissue sections of stretched, sub-rupture tendon were imaged with an Agilent Cary 670 interferometer and 620 IR microscope, equipped with a 64 × 64 Focal Plane Array Mercury Cadmium Telluride (MCT) detector. High magnification optics (1.1 μm × 1.1 μm pixels, 15×, 0.62NA objective) [25] were used to explore collagen orientation within intact tissue. All spectra were acquired as averages of 128 scans, 4 cm^−1^ spectral resolution, ratioed against a background of 512 scans acquired on a clean region of the salt window. Spectral acquisition and data processing were performed with the ResolutionsPro^TM^ FTIR spectroscopy software (Ver. 5.3.01964, Agilent Technologies Inc., Santa Clara, CA, USA).

O-PTIR spectra and images of tissue and of fibrils were collected on a mIRage+R™ Infrared and Raman microscope (Photothermal Spectroscopy Corp, Santa Barbara, CA, USA). Samples were presented on CaF_2_ windows and glass microscope slides and measured in reflectance mode. The system was equipped with a pulsed, broadly tunable high-power QCL covering 1800–800 cm^−1^ as the pump beam (IR source) and a 532 nm continuous wave (CW) probe beam laser. Upon IR frequency-dependent absorption, the photothermal expansion and refractive index changes are detected via lock-in amplifier detection of variations in the reflected probe beam (532 nm) intensity as a function of IR wavelength sweeping. Consequently, the spatial resolution of the IR spectra is now diffraction-limited by the wavelength of the probe beam, to about 350 nm.

The system was purged with dry nitrogen gas to remove water vapor interferences. Data were collected by focusing the sample using the visible image, which was collected with both the 10× visible objective (for sample overview) and the 40×, 0.78 NA reflective Cassegrain-style objective for both sample visualization and O-PTIR data collection. Background spectra were collected on aluminized polypropylene mounted on epoxy to provide an accurate power spectrum of the QCL. This was carried out once at the beginning of the day and used throughout; the QCL was set to 100% power, 100 kHz repetition rate, with a pulse width of 300 ns (3% duty cycle). The probe beam was set to 25% power. Twenty scans were co-added at 6 cm^−1^ spectral resolution, with a QCL scan rate of 100 cm^−1^/s. Sample spectra were collected at 79% QCL power, 100 kHz repetition rate, 300 nm pulse width (3% duty cycle). The probe beam was also set at 79% power. Three scans were co-added at 6 cm^−1^ spectral resolution with a QCL scan rate of 100 cm^−1^/s. All sample spectra were automatically normalized to the collected background spectrum. IR images were collected at discrete frequencies as specified in the results section. Images were collected with 100 nm pixel sizes at a line scan rate of 1 Hz, with 10 Hz retrace.

Nano-FTIR data were acquired with a neaSNOM (neaspec GmbH, Munich-Haar, Germany) microscope at beamline 2.4 and with the synchrotron infrared nanospectroscopy (SINS) system [16] at beamline 5.4, ALS, according to protocols detailed previously [7]. With SINS, spectra were recorded with a modified commercial rapid-scan FTIR spectrometer (Nicolet 6700, Thermo-Scientific, Madison, WI, USA), with an asymmetric Michaelson interferometer. The second order amplitude (s(ω)) and phase (φ(ω)) spectra of the backscattered light were recorded at locations on (sample) and off (reference) the fibrils as a function of frequency, ω. For each sample and reference spectrum, 512 scans were co-added, with a spectral data spacing of 8 cm^−1^. Spectra were processed with a fast Fourier-transform analysis suite (Nanospectroscopy Data Analysis (V.1, Advanced Light Source, Berkeley, CA, USA). Data collection with the commercial neaSNOM system at beamline 2.4 was conducted similarly, within the standard operating protocols of the system, synchrotron IR illumination and MCT detector. Finally, spectra were acquired with a neaSNOM microscope and 4.5–15 μm nano-FTIR laser illumination. Spectra were analyzed with the neaPLOT^TM^ software package (Ver. 1.9.702, neaspec GmbH, Munich-Haar, Germany). AFM and s-SNOM image data were processed in Gwyddion (Ver. 2.51, Brno, Czechia)) [29].

For all nano-FTIR spectra, whether acquired with synchrotron source IR or nano-FTIR laser illumination, the sample signal was normalized against a signal obtained from a clean location on the AuSi substrate. The gold- or platinum-coated AFM tips used in these experiments are often provided on a polydimethylsiloxane (PDMS) gel substrate that can lead to slight contamination of the tip. Even when transported in gel-free packaging, some contamination may occur in the manufacturing process. As a consequence, PDMS appears in the reference spectrum, observed as negative peaks in the sample spectra.

For post-processing of near-field data, the second order amplitude, s_2_(ω), and phase φ_2_(ω) spectra of the demodulated complex-valued scattering coefficient σ were recorded, and the imaginary part, which corresponds to the near-field IR absorption, was calculated [14,17] according to the relationship:

Im[σ_2_(ω)] = Im[s_2_(ω) e^i[φ2(ω)]^] (1)

The near-field nano-FTIR absorbance spectrum was obtained by normalizing the signal on a fibril against a signal from on a flat, non-absorbing surface location on the gold-coated wafer:

nano-FTIR spectrum = [s_2_(fibril, ω)/s_2_(Au, ω)] × [sin [φ_2_(fibril) − φ_2_(Au)]] (2)

Spectra from all sources were exported in spc file format for common display purposes.

## 5. Conclusions

We have used three different techniques to obtain polarized IR spectra of collagen in intact tendon and as isolated fibrils. The highly linearly polarized IR laser source in the FF O-PTIR yielded spectra of intact tendon that closely matched those obtained from IR polarized FF-FTIR FPA, and the expected dichroic behavior of spectra with respect to fiber orientation was easily displayed. FF-FTIR with FPA allows for the rapid survey of large areas of intact tissue, with or without polarization, though spatial resolution is wavelength-dependent across the 5–10 μm spectral range. With O-PTIR, individual spectra could be acquired rapidly (30s), with equally good SNR, from tendons mounted on salt windows or glass microscope slides. An obvious advantage of obtaining spectra from samples on glass is that subsequent histochemical or immunohistochemical analysis could be performed.

Acquisition of FF-FTIR FPA data from individual fibrils was not feasible; however, in this first demonstration of FF O-PTIR spectra and images of a biopolymer, good data were obtained from fibrils of ~500 nm diameter. Nano-FTIR spectroscopy gave access to the smallest feasible voxel, and from fibrils of 100 to 300 nm diameter. The O-PTIR spectra with perpendicular orientation were an excellent match for nano-FTIR spectra from the smaller fibrils, obtained under synchrotron infrared light and with nano-FTIR lasers.

Relative intensities and band profiles changed between parallel and perpendicular orientations of the fibers and fibrils to polarized IR light, as expected, but distinct differences were noted among the different samples and techniques. For the intact tendon, with IR light polarized parallel to fiber direction, the Amide II band intensity increased relative to the Amide I, again as expected, since the Amide II backbone mode is known to be well aligned with the fiber direction. Interestingly, the 1635 cm^−1^ shoulder in the Amide I band was more clearly defined in the O-PTIR spectra of intact tendon, in comparison to the FF-FTIR FPA results. This shoulder is often described as being mainly due to the C=O modes of the glycine residue. We conclude that the dipole moment change for this mode is more closely aligned with the molecular backbone. The increased definition of this band in the O-PTIR compared to FF-FTIR FPA spectra is thought to be due to the greater spatial resolution, with O-PTIR providing for a more pure local spectrum. The larger voxel probed with FF-FTIR FPA is averaged across more variations in fiber orientation, and could include additional, non-collagen materials. The Amide I and II bands were significantly narrower in fibril spectra compared to those from the intact tendon. The low energy shoulder at 1635 cm^−1^ was barely discernible against the high energy band at 1660 cm^−1^ whether from perpendicular O-PTIR or in the smallest fibrils with nano-FTIR. The collagen fibrils have radial symmetry at this length scale, and the electrical field is parallel to the AFM tip in s-SNOM nano-FTIR; these spectra were also effectively perpendicular. Only the FF O-PTIR could deliver spectra of fibrils with polarization parallel to the fibril orientation. The Amide II band was seen to be narrower than in the intact tendon, probably owing to greater orientation purity, while the 1660 and 1635 cm^−1^ maxima were again more clearly resolved. This is the first demonstration that O-PTIR and nano-FTIR spectra give confirmatory and complementary results on collagen fibrils with diameters from 100 to 500 nm. We conclude that these spectra represent reliable profiles for control collagen type I fibrils; taken together, these results provide a broad basis for further studies of collagen in biological samples.
